# Recalcitrant Hailey–Hailey disease in a 55-year-old Filipino woman successfully treated with 5-fluorouracil and excimer lamp phototherapy: A case report

**DOI:** 10.1016/j.jdcr.2026.04.059

**Published:** 2026-05-06

**Authors:** Althea Julie W. Pabico, Elisa Rae L. Coo, Elizabeth Amelia V. Tianco, Zharlah Gulmatico-Flores

**Affiliations:** Department of Dermatology, Jose R. Reyes Memorial Medical Center, Manila, Philippines

**Keywords:** benign familial pemphigus, excimer lamp, Hailey–Hailey disease, phototherapy, 5-fluorouracil

## Introduction

Hailey–Hailey disease (HHD) or familial benign pemphigus is a rare genodermatosis that is characterized by recurrent vesicles, erosions, and fissures in flexural areas. In this autosomal dominant bullous dermatosis, lesions are localized especially to the axillary and inguinal regions. No gender or ethnic group predilection is noted, and it usually presents in the second to fourth decades of life. The disease is caused by mutations in the *ATP2C1* gene.

Its diagnosis is based on lesion morphology and location, family history, and the characteristic brick-wall appearance of the epidermis on histopathology. There is no established curative therapy for HHD. Its therapeutic approach involves the control of exacerbating factors, secondary infections, and cutaneous inflammation.^1^ Because of the rarity of the disease, evidence of efficacy is mainly based on small observational studies, case reports, and clinical experience. Based on Philippine data, there have only been 20 cases recorded in the past 8 years (2014-2022).^1^ In this report, we present a case of HHD in a 55-year-old Filipino woman responding well to 5-fluorouracil (5-FU) cream 3% and excimer phototherapy after being on multiple treatment strategies.

## Case report

**Fig 1:**
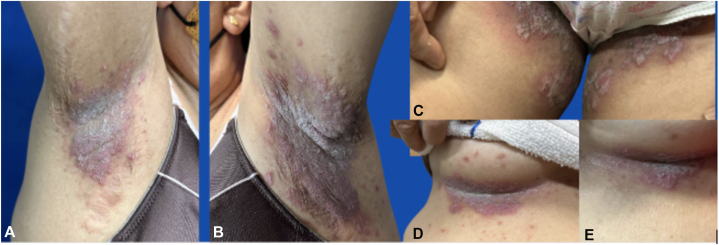
Erythematous plaques topped with white scales on the (**A**) left and (**B**) right axillae, (**C**) medial thighs, and (**D, E**) inframammary folds.

**Fig 2:**
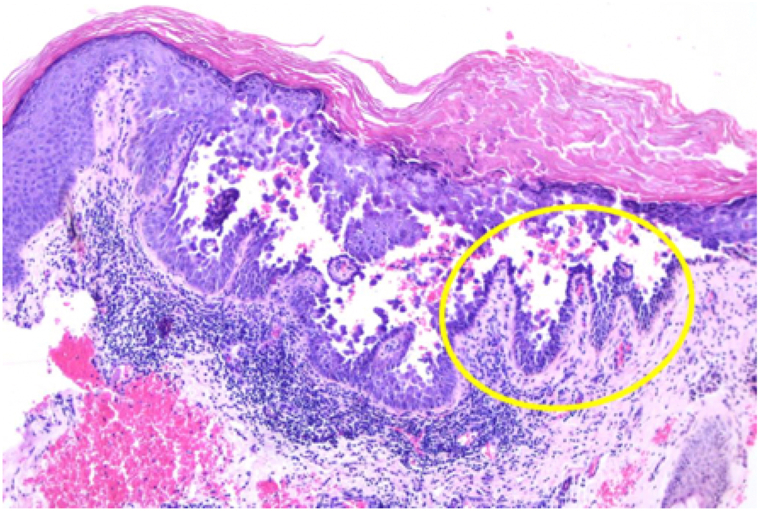
Histopathology shows the characteristic “dilapidated brick-wall” appearance. The *yellow circle* highlights the area of suprabasal acantholysis demonstrating the characteristic “dilapidated brick-wall” appearance.

A 55-year-old Filipino woman presented with a 4-year history of recurrent painful erosive plaques involving the bilateral axillae, inframammary folds, and inguinal areas. Flares were associated with malodor, fissuring, and significant discomfort, impairing daily activities. Family history shows similar lesions among the father and her 2 sisters. Physical examination showed erythematous, macerated plaques with erosions and fissures in intertriginous sites (Fig 1). Histopathologic examination revealed suprabasal acantholysis (Fig 2) consistent with HHD.

Prior treatments resulted in partial or transient improvement and included topical modalities of corticosteroids, antifungals, tacrolimus, topical and systemic antibiotics, and oral methotrexate. The patient underwent 1 session of carbon dioxide laser therapy but discontinued due to pain. Narrow-band UV-B phototherapy was attempted but was stopped after worsening of lesions.

**Fig 3:**
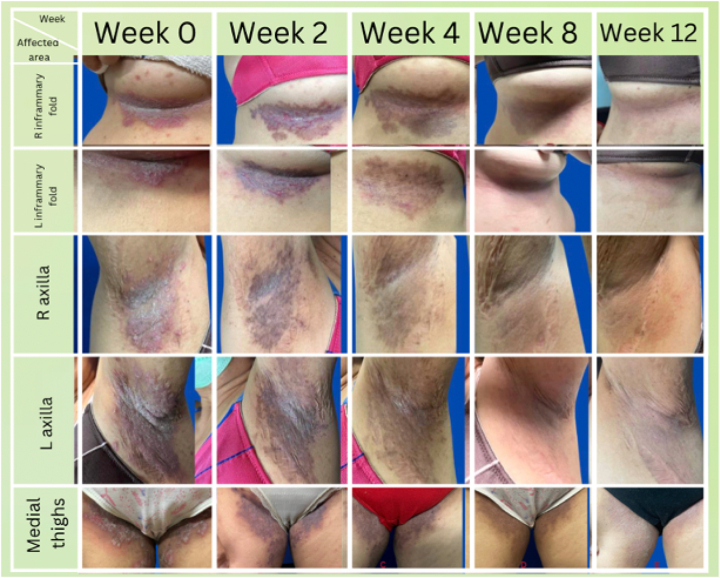
The patient’s lesion from weeks 0, 2, 4, 8, and 12. *L*, Left; *R*, right.

Given refractory disease, combination therapy with topical 5-FU and targeted phototherapy was initiated. Topical 5-FU cream 3% was applied once daily for 1 week, then reduced to 3 times weekly. Excimer lamp phototherapy (Exciplex; Spectrumed, Inc) was administered 3 times weekly, starting at 100 mJ/cm^2^, with a 10% dose increase per session as tolerated. After 4 weeks (cumulative dose, 26,360 mJ/cm^2^), marked clinical improvement was observed, with decreased erythema, erosions, and pain. Continued improvement was noted at weeks 8 and 12 (Fig 3). Treatment was subsequently tapered to topical 5-FU twice weekly and excimer lamp phototherapy once weekly. The patient was eventually able to discontinue both therapies and remains in remission without recurrence to date.

## Discussion

HHD is a chronic relapsing acantholytic dermatosis caused by mutations in *ATP2C1*, leading to impaired calcium homeostasis and defective keratinocyte adhesion. Disease activity is triggered by friction, sweat, and heat, making treatment challenging for HHD. Moreover, there are only a given limited number of available randomized controlled trials that have shown effective resolution for the lesions.^2^

First-line therapies include topical corticosteroids, topical antimicrobials, and calcineurin inhibitors.^3^ Oral antibiotics can be useful in the treatment of HHD, especially when used in combination with topical treatments.^2^ Some regard botulinum toxin A also as first-line therapy for HHD by blocking the liberation of acetylcholine from the nerve terminals, and eventual reduction of sweat production by the eccrine glands.^4^ Second-line therapies include procedural modalities such as carbon dioxide laser therapy and dermabrasion; these have been reported to be effective but pain, downtime, and relapse after discontinuation remains a concern.^3^ Other alternative options include oral retinoids such as acitretin, etretinate, and oral immunomodulators can be considered.^2^

Topical 5-FU has been hypothesized as a potential therapeutic option for HHD, possibly through effects on epidermal differentiation and intracellular calcium regulation, thereby helping to compensate for impaired calcium pump activity. A patient with HHD was treated with topical 5-FU 5% cream 3 times weekly for 3 months, then once weekly for 3 months noted remission after 3 months, and no relapses in the following year.^5^

UV radiation, in contrast, is considered an exacerbating factor of HHD because it induces inflammatory cytokines and suppresses *ATP2C1* expression, leading to acantholysis. However, targeted UV-B therapy may also provide anti-inflammatory effects through reduction of T-lymphocytes and other immune mediators in the skin, and limited reports have described benefit in treatment-resistant HHD.^6^^,^^7^ A combination of retinoids and narrowband ultraviolet B was also reported in a patient who was unsuccessfully treated with multiple modalities.^8^ Although there are already a few reports in the literature of UV-B phototherapy for the treatment of recalcitrant HHD, we were only able to come across 1 study which used VTRAC (Strata Skin Sciences, Inc) excimer lamp phototherapy alone for treatment and after 8 months of treatment, the frequency of irradiation was successfully reduced to every second week, with no exacerbation of HHD.^4^

To the best of our knowledge, this is the first reported case demonstrating successful treatment of HHD using a combination of topical 5-FU and excimer lamp phototherapy. The favorable outcome observed in this patient suggests that a potential synergistic effect between these modalities may provide complementary mechanisms that enhance clinical efficacy compared with monotherapy. This therapeutic approach may represent a promising alternative for patients with refractory HHD, although further studies are needed to better define its safety, durability of response, and broader applicability.

## Conclusion

This case demonstrates that combination therapy with topical 5-FU and excimer lamp phototherapy may be an effective option for patients with HHD refractory to conventional treatments. This approach may offer a targeted and synergistic alternative in difficult-to-treat cases.

## Conflicts of interest

None disclosed.
